# A Study to Evaluate the Effect of Alpha-Lipoic Acid on Neuropathic Symptoms in Diabetic Neuropathy Patients on Gabapentin or Pregabalin

**DOI:** 10.7759/cureus.70299

**Published:** 2024-09-27

**Authors:** Usharani Pingali, Sireesha Kammila, Padmaja Mekala, Sireesha Yareeda, Sravanasandya Penugonda

**Affiliations:** 1 Clinical Pharmacology and Therapeutics, Nizam's Institute of Medical Sciences, Hyderabad, IND; 2 Neurology, Nizam's Institute of Medical Sciences, Hyderabad, IND

**Keywords:** alpha lipoic acid, diabetic peripheral neuropathy, neurone specific enolase, neuropathy total symptom score 6, oxidative stress, vibration perception threshold

## Abstract

Introduction

Chronic hyperglycemia is a key factor in the development of diabetic peripheral neuropathy (DPN), contributing significantly to the progression of this condition through the induction of oxidative stress. Elevated blood glucose levels lead to increased production of reactive oxygen species (ROS), which cause damage to neuronal cells and exacerbate neuropathic symptoms. Alpha-lipoic acid (ALA) is a potent antioxidant that neutralizes ROS and reduces oxidative damage. By addressing the mechanisms of neuropathy, ALA mitigates the effects of chronic hyperglycemia through antioxidant regeneration, inflammation reduction, and endothelial function improvement. As a result, ALA is emerging as a promising intervention for managing oxidative stress and inflammation in DPN.

Methods

Prospective, double-blind, placebo-controlled study, a total of 52 DPN subjects on gabapentin or pregabalin were randomly assigned to either ALA 600 mg oral once daily or placebo oral once daily for 12 weeks. Treatment outcome was assessed using vibration perception threshold (VPT), Neuropathy Total Symptom Score 6 (NTSS-6), quality of life (QOL) assessed by 12-Item Short Form Health Survey (SF-12) Questionnaire, oxidative stress biomarkers (malondialdehyde (MDA), nitric oxide (NO), and glutathione (GSH)) and inflammatory biomarker (high-sensitivity C-reactive protein (hs-CRP)) at baseline and every visit (four, eight, and 12 weeks). Neuron-specific enolase (NSE) levels were done at baseline and 12 weeks. Glycated hemoglobin (HbA1C) and safety parameters were done at screening and 12 weeks. Compliance with the study medication was assessed by pill count. Reported adverse drug reactions were recorded.

Results

A total of 52 subjects (males = 22; females = 30) with a mean age of 55.63 ± 7.5 years were randomized to receive either ALA or placebo. A significant reduction in VPT was observed with ALA at 12 weeks compared to baseline (from 26.92 ± 3.58 to 22.14 ± 1.94; p < 0.0001). Secondary parameters like NTSS-6, QOL by SF-12 questionnaire, NSE levels, oxidative biomarkers levels, and inflammatory biomarkers showed significant improvement with ALA compared to placebo. No serious adverse events were reported.

Conclusion

ALA demonstrated a protective effect against DPN by its antioxidant and anti-inflammatory effects and was found to have good safety.

## Introduction

Diabetic peripheral neuropathy (DPN) is a common microvascular complication that occurs in patients with diabetes mellitus (DM) [1]. DM is caused by abnormal insulin secretion or function, resulting in hyperglycemia. Chronic hyperglycemia leads to oxidative stress and the generation of free radicals, resulting in microvascular and macrovascular diseases and complications [2].

DPN is characterized by varying degrees of sensory loss, leading to poor recognition of foot trauma [3]. Early diagnosis of DPN is important, and measurement can be done using objective tests and specific biomarkers. Vibration perception threshold (VPT) is a non-invasive diagnostic test for evaluating DPN, even in the early stages. It is a quick, inexpensive, and accurate screening procedure for assessing high-risk patients [4]. Research by Young et al. [5] found that patients with VPT > 25 V had a sevenfold greater risk of foot ulceration compared to those with VPT < 15 V.

Neuron-specific enolase (NSE) is a newly emerging biomarker of peripheral neuropathy in diabetes. After nerve injury, NSE is readily released into the cerebrospinal fluid and blood. Serum NSE levels were significantly elevated in diabetic subjects with neuropathy [6]. Animal models with streptozotocin-induced diabetes also showed increased levels of NSE due to hyperglycemia [7].

Current guidelines recommend tricyclic agents, serotonin-norepinephrine reuptake inhibitors, or gamma-aminobutyric acid analogs as first-line agents for neuropathic pain, followed by opioids and topical treatments. Pregabalin and gabapentin are commonly used but can cause side effects [8,9]. These drugs do not affect the underlying disease pathogenesis. Antioxidants, which can target multiple pathways involved in neuropathy pathogenesis, play a promising role in preventing disease progression [10].

Alpha-lipoic acid (ALA) is a potent antioxidant known to prevent pancreatic beta cell destruction, increase glucose uptake, and delay the development of DPN. The neuroprotective and pain-relieving effects of ALA therapy have been reported in the literature [11]. The Symptomatic Diabetic Neuropathic Study 2 (SYDNEY 2) trial suggests that treatment using 600 mg of ALA orally once daily for five weeks reduces the chief symptoms of DPN, including pain, paresthesia, and numbness to a clinically meaningful degree [12].

In this study, ALA was evaluated for its protective role in DPN. The primary objectives were to study the effect of ALA on VPT and the Neuropathy Total Symptom Score 6 (NTSS-6). The secondary objectives were to study the effect of ALA on oxidative stress biomarkers, inflammatory biomarkers high-sensitivity C-reactive protein (hs-CRP), quality of life (QOL) by 12-Item Short Form Health Survey (SF-12), and NSE, and to assess the safety and tolerability of ALA.

## Materials and methods

This was a prospective, randomized, double-blind, placebo-controlled, parallel-group study. This study was conducted in the Department of Clinical Pharmacology and Therapeutics in collaboration with the Department of Neurology at a tertiary care hospital after approval by the Institutional Ethics Committee and also registered in Clinical Trial Registry India (CTRI/2021/10/037068) as protocol entitled “A study to evaluate the effect of ALA on neuropathy symptoms and inhibition of platelet aggregation in diabetic neuropathy patients on gabapentin/pregabalin.” This study was done in two parts. This is the first part of the data regarding neuropathic symptoms that is now being submitted. The study was conducted under the Declaration of Helsinki and Good Clinical Practice Guidelines issued by the Government of India.

Voluntary written informed consent was taken from all the study participants before enrollment. The study population included symptomatic DPN patients with a VPT value of more than 15 V of either gender, taking a stable dose of either gabapentin 300 mg BD (twice daily) or pregabalin 75 mg BD for the past three months, aged between 30 and 65 years, patients with DM duration > 10 years, on a stable dose of antidiabetic medication (allowed medication single drug or drug combination of metformin, sulphonylureas or DDP4 inhibitors for the past three months), glycated hemoglobin (HbA1C) <10% and serum creatinine < 2 mg/dL. Subjects with a history of hypersensitivity to ALA, any other neuropathic pain conditions, receiving other antioxidants, pregnant and lactating women, and clinically significant skin diseases, including foot ulcers, corns, and plantar fasciitis, were excluded from the study. The screening was done by taking a medical history, clinical examination, and necessary laboratory investigations, and eligible subjects were randomized. All the eligible subjects were asked to come to the department in a fasting state.

At the baseline visit (zero weeks), history and physical examination, including vitals, were done. Baseline NTSS-6 and QOL assessment by SF-12 questionnaire were assessed, and the VPT was measured using a biothesiometer. A blood sample was collected at the baseline visit for estimation of fasting blood sugar (FBS), postprandial blood sugar (PPBS), malondialdehyde (MDA), nitric oxide (NO), glutathione (GSH), hs-CRP, and NSE. The subjects were randomized by simple randomization into two groups by computer-generated random numbers. One group received ALA 600 mg once daily, and another group received an identical placebo in a double-blind manner. The treatment duration was 12 weeks. All the subjects were on stable doses of antidiabetic medication and gabapentin or pregabalin throughout the study. Follow-up visits were at four, eight, and 12 weeks. NTSS-6 score, QOL assessment, VPT assessment, oxidative stress biomarkers (NO, MDA, and GSH), hs-CRP, and blood sugar levels (FBS, PPBS) were done at four, eight, and 12 weeks. NSE levels were measured at baseline and 12 weeks. All the patients were monitored for safety and tolerability till 12 weeks. The levels of NO, MDA, and GSH were estimated using a spectrophotometer. hs-CRP levels were estimated using an immunoturbidimetry method using Cobas kit (Roche Diagnostics GmbH, Mannheim, Germany). NSE levels were estimated by using an enzyme-linked immunosorbent assay (ELISA) kit. FBS and PPBS levels were estimated using a glucometer.

Statistical analysis was performed using GraphPad Prism version 9.0.2 (Graph Pad Prism version 9.0.2) and Microsoft Excel (Microsoft Corp., Redmond, WA). Data were presented as mean ± standard deviation. Safety data were represented as numbers. Comparison within the group was performed using a paired t-test, and comparison between the groups was performed using an unpaired t-test. A p-value < 0.05 is considered statistically significant. The primary efficacy parameter was the change in VPT from baseline, and the secondary efficacy parameters were changes in NTSS-6 score, MDA, NO, GSH, hsCRP, NSE, and QOL. All the subjects who have completed at least one post-baseline efficacy assessment were considered for efficacy analysis. All randomized subjects were included for safety analysis and intention to treat the population. The sample size was calculated based on a study by El-Nahas et al. [13]. Taking the mean difference of VPT values between the ALA-treated and placebo groups as 5.1 V with a standard deviation of 6.5, 50 subjects were required to reject the null hypothesis. Considering a 10% dropout rate and a 10% screen failure rate, a total of 60 subjects were screened. The power of the study was 80%, and the type I error probability was 0.05.

NTSS-6 score was taken at times specified in the scheduled protocol. Symptoms were graded based on the experience of the subject during the past 24 hours. Each symptom question can be assigned a maximum score of 3.66 points. The NTSS-6 total score was the sum of the six individual symptom scores and had a maximum of 21.96 points. The score was given based on the subject's response to the symptom question. Reduction in NTSS-6 score denotes improvement in neuropathy symptoms.

VPT was measured using a biothesiometer at specified times. The probe was first applied to the subject's hand to demonstrate the vibration sensation. Then, it was applied perpendicularly to six points on each foot (great toe, base of first, third, and fifth metatarsals, instep, and heel), with voltage gradually increasing from 0 to 50 V. Subjects reported when they first felt the vibration. VPT was recorded three times at each site, with the probe removed between readings. If the Coefficient of variation (CV) exceeded 15%, additional readings were taken. The average of these recordings constitutes the final VPT value.

## Results

**Figure 1:**
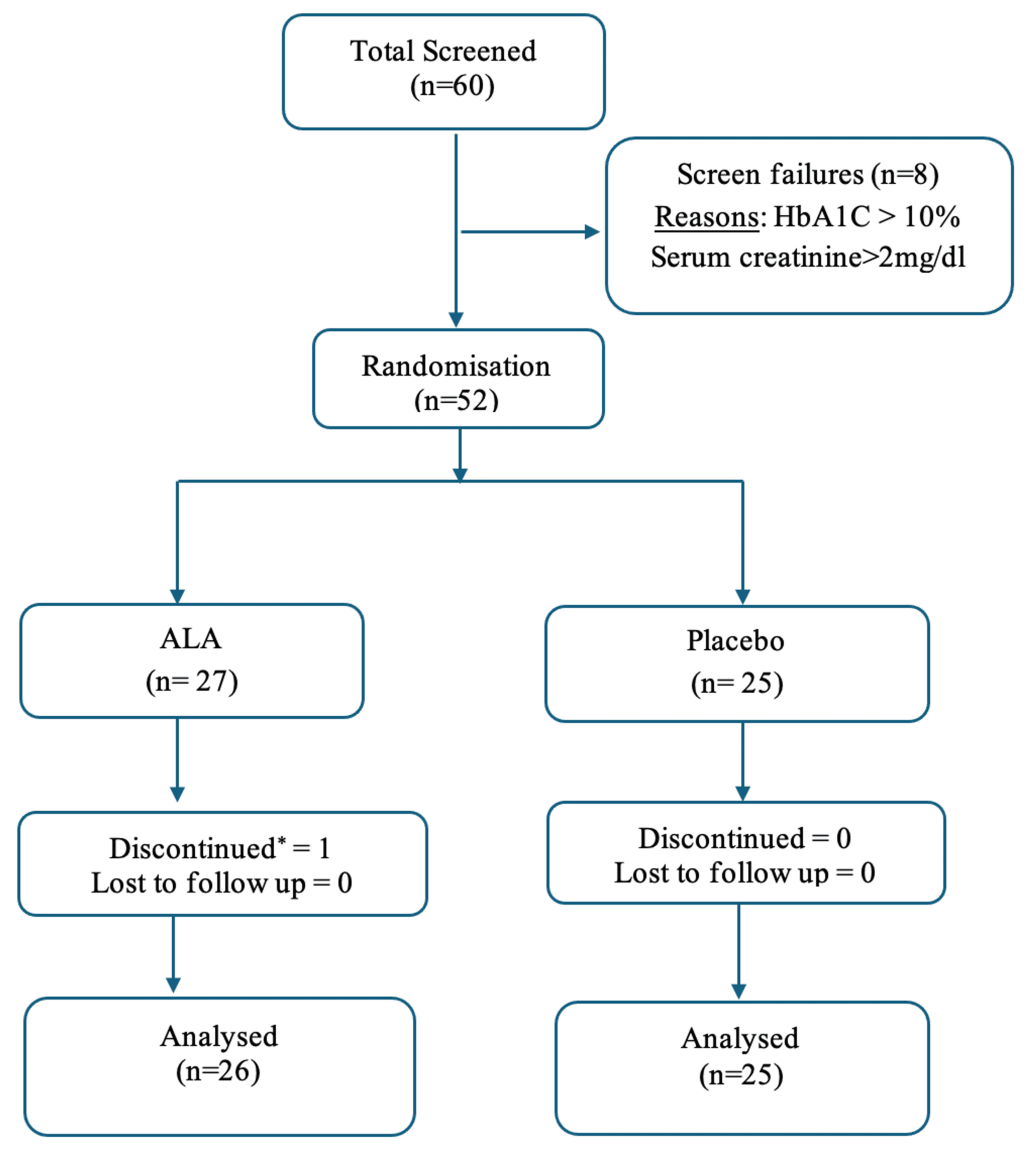
Participant flow diagram ALA - alpha-lipoic acid, HbA1C - glycated hemoglobin *Relocated to another city

A total of 60 subjects were screened, and 52 eligible subjects were randomized (ALA group, n = 27; placebo group, n = 25). Eight were excluded as they did not meet the eligibility criteria. Out of 52 randomized, 51 subjects were included in the final analysis. A participant flow diagram is presented in Figure 1. Of the enrolled 52 subjects, females were 30, and males were 22, with a mean age of 55.63 ± 7.5 years. The demographic and baseline characteristics of the study population presented as mean ± standard deviation are presented in Table 1 and Table 2.

**Table 1:** Demographic details of study subjects ALA - alpha-lipoic acid, BMI - body mass index

S. no.	Parameter	ALA (n = 26)	Placebo ( n = 25)	p-value
1	Age (years)	55.34 ± 6.4	55.12 ± 7.72	0.64
2	Male:female ratio	11:15	11:14	
3	BMI (kg/m^2^)	25.9 ± 2.5	24.04 ± 2.31	0.06

**Table 2:** Baseline characteristics of study subjects ALA - alpha-lipoic acid, FBS - fasting blood sugar, GSH - glutathione, HbA1C - glycated hemoglobin, hs-CRP - high-sensitivity C-reactive protein, MDA - malondialdehyde, NO - nitric oxide, NSE - neuron-specific enolase, NTSS-6 - Neuropathy Total Symptom Score 6, PPBS - postprandial blood sugar, SF-12 - 12-Item Short Form Health Survey, VPT - vibration perception threshold

S. no.	Parameter	ALA (n = 26)	Placebo (n = 25)	p-value
1	VPT (V)	26.92 ± 3.58	25.69 ± 2.34	0.15
3	SF-12	33.65 ± 3.01	33.44 ± 2.38	0.78
4	NTSS-6 score	11.61 ± 1.43	11.15 ± 1.46	0.27
5	MDA (nM/ml)	4.79 ± 0.64	5.01 ± 0.76	0.26
6	GSH (µM/L)	514.12 ± 22.31	512.28 ± 40.71	0.84
7	NO (µM/L)	33.68 ± 4.99	30.99 ± 5.79	0.08
8	hs-CRP (mg/L)	5.88 ± 1.77	5.29 ± 1.55	0.21
9	NSE (µg/L)	16.23 ± 6.26	15.17 ± 3.81	0.47
10	HbA1C (%)	7.80 ± 0.67	7.76 ± 0.90	0.86
11	FBS (mg/dL)	123.76 ± 13.26	122.60 ± 12.70	0.75
12	PPBS (mg/dL)	164.65 ± 21.65	162.56 ± 19.67	0.72
13	Mean duration of DM	12.8 ± 2.26	13.2 ± 1.69	0.8

**Figure 2:**
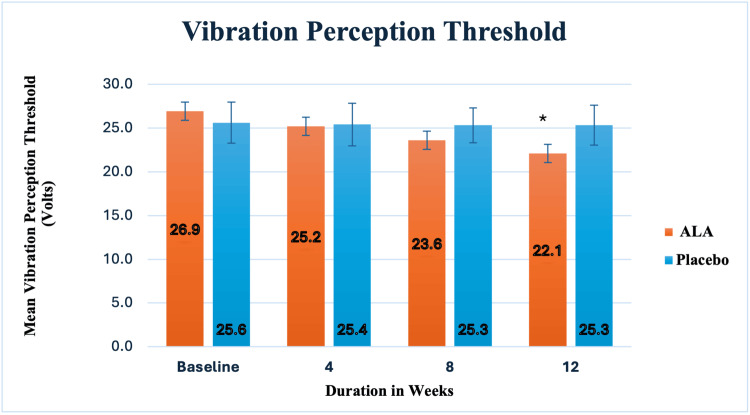
Showing the mean values of vibration perception threshold at baseline, four, eight, and 12 weeks, respectively, in the ALA and placebo groups ALA - alph-lipoic acid *p = 0.00002, compared to placebo

The study variables followed a normal distribution, as confirmed by the Shapiro-Wilk and Kolmogorov-Smirnov tests. The groups were homogeneous and comparable at baseline, with p-values greater than 0.05. The primary outcome measure was the comparison of mean VPT values at baseline, four, eight, and 12 weeks, both within and between the groups. ALA demonstrated a significant reduction in VPT at four, eight, and 12 weeks compared to baseline. In contrast, the placebo group showed no significant change in VPT at any of these time points. Additionally, ALA resulted in a significant reduction in VPT values at eight weeks (p = 0.01) and 12 weeks (p = 0.00002) compared to the placebo group, as shown in Figure 2.

**Figure 3:**
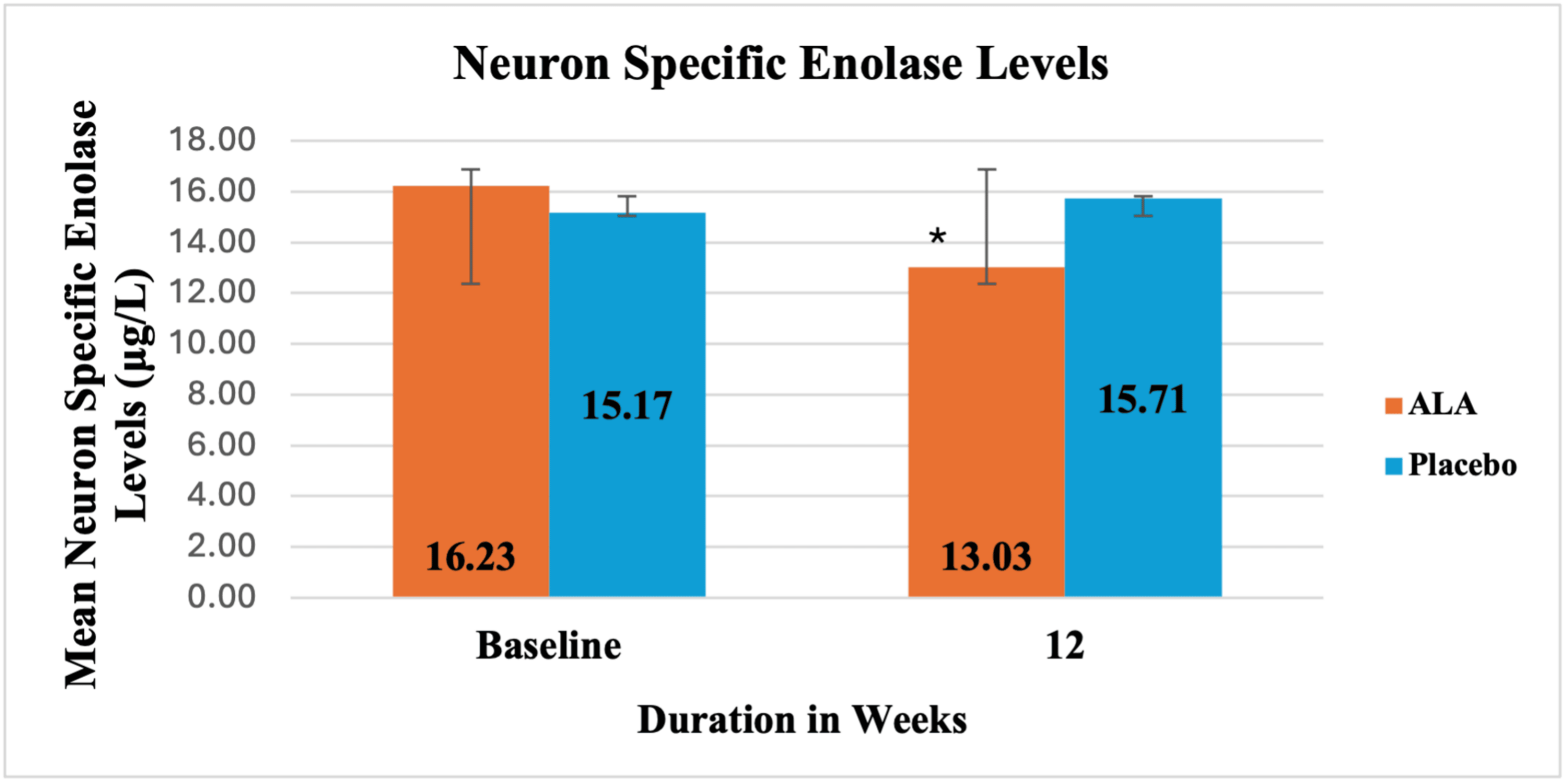
Showing the mean values of neuron-specific enolase levels at baseline and 12 weeks, respectively, of ALA and placebo groups ALA - alpha-lipoic acid *p = 0.04, compared to placebo

The secondary outcome measures included evaluating the effects of ALA versus placebo on the NTSS-6 score, SF-12 questionnaire, oxidative stress biomarkers, hs-CRP, NSE levels, and overall safety, as presented in Table 3. The secondary outcome measure was the comparison of mean values of NTSS-6 score at baseline, four, eight, and 12 weeks within the group and between the groups. There was a significant reduction in the mean NTSS-6 score at four, eight, and 12 weeks with ALA and at 12 weeks with placebo compared to baseline. However, there was no significant change in the mean NTSS-6 score with placebo at four and eight weeks compared to baseline. ALA showed a significant reduction in NTSS-6 score at eight (p = 0.005) and 12 weeks (p = 0.0003) compared to placebo (Table 3). ALA showed significant improvement in the SF-12 questionnaire at eight and 12 weeks compared to placebo. ALA showed a significant reduction in MDA levels at four, eight, and 12 weeks compared to placebo. ALA showed a significant increase in NO levels at the 12 weeks and GSH levels in the eight and 12 weeks compared to Placebo. ALA showed a significant reduction in hs-CRP levels at the four, eight, and 12 weeks compared to placebo. ALA demonstrated a significant reduction in the NSE levels compared to placebo at 12 weeks, as shown in Figure 3. Both ALA and placebo showed a significant reduction in FBS levels and PPBS levels at four, eight, and 12 weeks (p < 0.05), and a significant reduction in HbA1C compared to baseline was observed at 12 weeks (p< 0.05), as represented in Table 3.

**Table 3:** Showing the means and mean % change of secondary outcome measures in ALA and placebo groups ALA - alpha-lipoic acid, FBS - fasting blood sugar, GSH - glutathione, HbA1C - glycated hemoglobin, hs-CRP - high-sensitivity C-reactive protein, MDA - malondialdehyde, NA - not applicable, NO - nitric oxide, NS - not significant, NTSS-6 - Neuropathy Total Symptom Score 6, PPBS - postprandial blood sugar, SF-12 - 12-Item Short Form Health Survey

Parameter	Weeks	ALA (n = 26)	Mean % change from baseline	Placebo (n = 25)	Mean % change from baseline	p-value
NTSS-6 score	Baseline	11.61 ± 1.43	NA	11.15 ± 1.46	NA	NS
	Four weeks	10.48 ±1.42	-9.72 ± 5.17	10.90 ± 1.53	-2.21 ± 6.69	NS
	Eight weeks	9.48 ± 1.63	-18.37 ± 9.46	10.58 ± 1.61	-2.97 ± 8.32	0.005
	12 weeks	8.80 ± 1.75	-24.23 ± 11.61	10.42 ± 1.81	-3.98 ± 8.04	0.0003
SF-12 questionnaire	Baseline	33.65 ±3.01	NA	33.44 ± 2.38	NA	NS
	Four weeks	34.81± 2.56	3.65 ± 4.21	33.56 ± 2.84	0.36 ± 4.89	NS
	Eight weeks	36.08± 2.16	7.62 ± 6.21	34.2 ± 3.45	2.27 ± 7.39	0.02
	12 weeks	37.54 ± 1.94	12.08 ± 7.09	34.96 ± 3.86	4.59 ± 9.40	0.004
MDA (nM/mL)	Baseline	4.79 ± 0.64	NA	5.01 ± 0.76	NA	NS
	Four weeks	4.57 ± 0.69	-4.62 ± 6.66	5.06 ± 0.73	1.29 ± 9.01	0.02
	Eight weeks	4.27 ± 0.82	-11.14 ± 10.01	5.00 ± 0.79	0.07 ± 10.86	0.002
	12 weeks	4.05 ± 0.84	-15.73 ± 11.57	4.92 ± 0.80	-1.84 ± 5.72	0.0004
GSH (µM/L)	Baseline	514.12 ± 22.31	NA	512.28 ± 40.71	NA	NS
	Four weeks	524.01 ± 29.99	1.93 ± 3.89	510.30 ± 32.03	0.01 ± 7.42	NS
	Eight weeks	531.96 ± 31.46	3.51 ± 4.99	505.08 ± 35.54	-0.70 ± 7.27	0.007
	12 weeks	561.18 ± 42.53	9.19 ± 3.36	513.05 ± 37.22	0.65 ± 6.57	0.00006
NO (µM/L)	Baseline	33.68 ± 4.99	NA	30.99 ± 5.79	NA	NS
	Four weeks	33.92 ± 4.96	0.77 ± 1.20	31.24 ± 5.64	2.87 ± 9.28	NS
	Eight weeks	34.43 ± 4.99	2.34 ± 2.59	31.60 ± 5.14	2.64 ± 7.25	NS
	12 weeks	35.08 ± 4.72	4.38 ± 2.98	32.02 ± 6.43	3.14 ± 7.77	0.03
hs-CRP (mg/L)	Baseline	5.88 ± 1.77	NA	5.29 ± 1.55	NA	NS
	Four weeks	5.19 ± 1.59	-10.59 ± 15.91	5.20 ± 1.48	1.57 ± 5.36	NS
	Eight weeks	4.67 ± 1.45	-20.03 ± 10.54	5.23 ± 1.51	-0.90 ± 5.35	NS
	12 weeks	4.38 ± 1.73	-24.39 ± 25.42	5.34 ± 1.63	0.88 ± 6.83	0.04
FBS (mg/dL)	Baseline	123.77 ±13.26	NA	122.60 ± 12.70	NA	NS
	Four weeks	116.27 ± 11.96	-5.7 ± 7.2	119.36 ± 13.88	-2.6 ± 5.3	NS
	Eight weeks	114.23 ± 12.00	-7.4 ± 7.0	118.40 ± 12.96	-3.2 ± 7.15	NS
	12 weeks	113.65 ± 10.66	-7.7 ± 8.3	115.84 ± 11.87	-5.2 ± 7.89	NS
PPBS (mg/dL)	Baseline	164.65 ± 21.65	NA	162.56 ± 19.67	NA	NS
	Four weeks	156.00 ± 12.89	-4.5 ± 8.3	159.84 ± 19.20	-1.6 ±3.75	NS
	Eight weeks	152.58 ± 13.66	-6.6 ± 7.69	156.04 ± 17.56	-3.8 ± 4.98	NS
	12 weeks	148.92 ± 14.20	-8.7 ± 9.23	154.20 ± 15.11	-4.7 ± 6.54	NS
HbA1C (%)	Baseline	7.8 ± 0.67	NA	7.76 ± 0.90	NA	NS
	12 weeks	6.96 ± 0.74	-10.5 ± 8.72	7.37 ± 0.72	-4.63 ± 6.93	NS

A total of nine adverse events were noted in 52 enrolled subjects. The most common adverse events reported were headache, followed by nausea, as shown in Table 4. All the adverse events were managed symptomatically without stopping treatment. Compliance was assessed by the pill-count method, and all subjects showed good compliance.

**Table 4:** Showing the incidence of adverse drug reactions in both groups ALA - alpha-lipoic acid

Adverse drug reaction	ALA (n = 27)	Placebo (n = 25)
Gastritis	0	1
Headache	0	3
Fever	0	2
Nausea	2	0
Diarrhea	1	0
Total	3	6

## Discussion

The primary aim of this study was to evaluate the effect of ALA on neuropathic symptoms in diabetic neuropathy (DN) patients who are also taking gabapentin/pregabalin. This is a randomized, double-blind, placebo-controlled study. We found that compared to placebo, the administration of ALA at a dosage of 600 mg once daily for 12 weeks resulted in significant improvement across multiple parameters. These included a reduction in VPT, NTSS-6, levels of NSE, hs-CRP, and MDA. Additionally, ALA treatment led to a significant increase in QOL (assessed by SF-12), levels of NO, and GSH. 

VPT has significantly aided in the early diagnosis of peripheral neuropathy. Previous studies have shown that VPT is an excellent tool for assessing mild-to-moderate DN, providing a quantifiable evaluation of peripheral nerve involvement. Our study found a significant reduction in VPT with ALA at four, eight, and 12 weeks compared to baseline. Between-group analysis showed a statistically significant reduction in VPT values at eight and 12 weeks. This improvement in VPT has important clinical implications for DN management. Interventions targeting sensory nerve function, such as ALA supplementation, may help to reduce complications and enhance the QOL in DN patients. Our findings align with those of El-Nahas et al. [13], who administered ALA at 600 mg twice daily for six months. Their study reported a significant reduction in VPT at one, three, and six months using a neurothesiometer. In our study, we observed a 5.49% reduction in VPT in the fourth week, comparable to El-Nahas et al.’s [13] study, where they observed a 5.7% reduction in VPT in one month. In our study, ALA was administered once daily, while in the other study, ALA was administered twice daily.

In our study, we observed a significant reduction in NTSS-6 scores with ALA at four, eight, and 12 weeks and with placebo at 12 weeks compared to baseline. El-Nahas et al. [13] reported similar findings in neuropathy symptom scores after one month. The other study by Ziegler et al. (Alpha-Lipoic Acid in Diabetic Neuropathy (ALADIN) study) [14] showed significant improvements in neuropathic symptoms after three weeks of ALA treatment, consistent with our results at four weeks. These findings suggest that ALA may provide rapid relief of neuropathy symptoms. The SYDNEY 2 trial [11] demonstrated that oral ALA significantly improved symptomatic diabetic polyneuropathy compared to placebo, while the Neurological Assessment of Thioctic Acid in Diabetic Neuropathy (NATHAN 1) trial [15] reported sustained benefits over four years, supporting ALA's long-term use in reducing neuropathic symptoms and improving nerve function. DPN profoundly impacts QOL, affecting physical, emotional, and social well-being. In our study, QOL assessed by the SF-12 questionnaire significantly improved with ALA at four, eight, and 12 weeks and with placebo at 12 weeks compared to baseline. Between-group analysis at eight and 12 weeks showed a significant difference in SF-12 scores. Ziegler et al. [16] reported improvements in various QOL indices in DPN patients treated with ALA in a meta-analysis. However, studies using the SF-12 questionnaire to assess the QOL in DN patients are limited.

In our study, neuronal damage was assessed using NSE levels, which showed a significant reduction with ALA at 12 weeks compared to baseline and placebo. NSE is predominantly found in neurons and plays a crucial role in glycolysis, the process of glucose breakdown for energy. It is used as a biomarker for neuronal damage because it is released into the bloodstream. Ghaib et al. [17] found higher NSE levels in diabetic patients with neuropathy compared to those without, suggesting a potential association between NSE and DPN. However, direct human studies on ALA's impact on NSE levels are lacking.

ALA exerts its antioxidant effects through multiple mechanisms, including scavenging free radicals, regenerating other antioxidants(such as glutathione and vitamins C and E), and chelating transition metals. These mechanisms collectively contribute to the reduction of oxidative stress markers like MDA, GSH, and NO. In the current study, the antioxidant effect was assessed by oxidative stress biomarker levels (MDA, NO, and GSH). It was observed that with ALA administration, MDA levels were significantly reduced at four, eight, and 12 weeks compared to baseline and placebo. Cristian et al. [18] conducted a study in which they administered ALA 600 mg once daily intravenously for 10 days, followed by oral ALA 600 mg once daily for 30 days in DN patients. They observed that ALA supplementation significantly reduced the levels of MDA. In our study, NO and GSH levels were significantly increased at four, eight, and 12 weeks with ALA compared to baseline. Between-group analysis at eight and 12 weeks revealed a significant increase in NO and GSH levels. A study by Heitzer et al. [19] demonstrated that ALA supplementation improved endothelium-dependent, NO-mediated vasodilation in diabetic patients, suggesting enhanced NO bioavailability. This improvement in NO levels may contribute to the vasodilatory effects observed in DN, potentially alleviating symptoms and improving blood flow to the nerves. Zembron-Lacny et al. [20] found that ALA supplementation led to a significant decrease in oxidative stress markers, including lipid peroxidation and increased levels of GSH. 

In our study, hs-CRP levels were done to demonstrate the anti-inflammatory effect of ALA. It was found that ALA significantly reduced hs-CRP levels at four, eight, and 12 weeks when compared to baseline, and at 12 weeks, the reduction was significant as compared to placebo. Similar to our findings, Serhiyenko et al. [21] found that ALA 600 mg once a day for three months significantly reduced hs-CRP levels in individuals with cardiac autonomic neuropathy compared to placebo. The most common adverse events were headache and nausea, with the medications being well tolerated. No serious adverse events occurred, and no subjects discontinued the study due to adverse events, indicating ALA's good safety profile. Common ALA side effects include mild gastrointestinal symptoms like nausea, vomiting, and diarrhea. ALA is generally considered safe and well-tolerated in treating DN.

## Conclusions

In this study, we found that ALA significantly reduced VPT and NTSS-6 scores, along with improvements in QOL. ALA also notably enhanced oxidative stress biomarkers (MDA, GSH, NO) and the inflammatory marker hs-CRP. Furthermore, neuronal damage was significantly reduced, as evidenced by a decrease in NSE levels. These findings suggest that ALA exerts a protective effect against DPN through its antioxidant and anti-inflammatory properties while demonstrating a favorable safety profile. ALA may be recommended as a potential adjuvant to standard care for preventing the progression of DPN. However, further research with a larger sample size and extended duration is necessary to confirm these results and optimize the clinical use of ALA.

## Appendices

SF-12: This survey asks for your views about your health. This information will help keep track of how you feel and how well you are able to do your usual activities. Answer each question by choosing just one answer. If you are unsure how to answer a question, please give the best answer you can.

1. In general, would you say your health is: 

·      Excellent

·      Very good 

·      Good

·      Fair

·      Poor 

The following questions are about activities you might do during a typical day. Does your health now limit you in these activities? If so, how much? 

2. Moderate activities such as moving a table, pushing a vacuum cleaner, bowling, or playing golf. 

·      Yes, limited a lot 

·      Yes, limited a little 

·      No, not limited at all 

3. Climbing several flights of stairs. 

·      Yes, limited a lot         

·      Yes, limited a little 

·      No, not limited at all 

During the past four weeks, have you had any of the following problems with your work or other regular daily activities as a result of your physical health? 

4. Accomplished less than you would like: 

YES                                                     NO

5. Were limited in the kind of work or other activities. 

YES                                                     NO 

During the past four weeks, have you had any of the following problems with your work or other regular daily activities as a result of any emotional problems (such as feeling depressed or anxious)? 

6. Accomplished less than you would like: 

YES                                                     NO   

7. Did work or activities less carefully than usual. 

YES                                                     NO 

8. During the past four weeks, how much did pain interfere with your normal work (including work outside the home and housework)? 

·      Not at all             

·      A little bit 

·      Moderately 

·      Quite a bit 

·      Extremely 

These questions are about how you have been feeling during the past four weeks. For each question, please give the one answer that comes closest to the way you have been feeling. How much of the time during the past four weeks... 

9. Have you felt calm and peaceful?                         

·      All of the time                 

·      Most of the time 

·      A good bit of the time 

·      Some of the time 

·      A little of the time 

·      None of the time 

10. Did you have a lot of energy?                           

·      All of the time                  

·      Most of the time 

·      A good bit of the time 

·      Some of the time 

·      A little of the time 

·      None of the time 

11. Have you felt downhearted and blue?         

·      All of the time                      

·      Most of the time 

·      A good bit of the time 

·      Some of the time 

·      A little of the time 

·      None of the time 

12. During the past four weeks, how much of the time have your physical health or emotional problems interfered with your social activities (like visiting friends, relatives, etc.)?                                                                              

·      All of the time                  

·      Most of the time 

·      Some of the time 

·      A little of the time 

·      None of the time 
